# Unravelling Distress in Psychotic‐Like Experiences: A Systematic Review of Non‐Clinical Populations

**DOI:** 10.1111/eip.70175

**Published:** 2026-05-03

**Authors:** Joachim Rockenschaub, Antonia Renner, Fabian Friedrich, Maximus Berger, Clemens Mielacher, Melanie Trimmel, Barbara Hinterbuchinger, Nilufar Mossaheb

**Affiliations:** ^1^ Clinical Division of Social Psychiatry, Department of Psychiatry and Psychotherapy Medical University of Vienna Vienna Austria; ^2^ Comprehensive Center for Clinical Neurosciences and Mental Health Medical University of Vienna Vienna Austria

**Keywords:** distress, general population, psychotic‐like experiences, risk factors

## Abstract

**Introduction:**

Psychotic‐like experiences (PLEs) are subthreshold psychotic phenomena that occur along the psychosis continuum and are frequently accompanied by distress. However, the factors underlying distress in individuals reporting PLEs—particularly within non‐clinical populations—remain insufficiently understood. This systematic review aims to synthesise the evidence on factors directly or indirectly associated with distress in PLEs among non‐clinical samples.

**Methods:**

A systematic search of Medline, Web of Science, Embase, and Cochrane (January 2000–March 2025) using the terms *psychotic‐like experiences* AND (*distress* OR *resilience* OR *burden* OR *coping* OR *adaptive behaviour*) identified 762 studies, of which 111 met inclusion criteria and were included in a narrative synthesis.

**Results:**

Distress related to PLEs can be differentiated into direct PLE‐related distress and indirect psychological distress. Factors associated with distress clustered into three domains: (i) Symptomatology—greater frequency, intensity, persistence and specific subtypes predict higher distress, as do comorbid depression, anxiety and suicidality. (ii) Psychological factors—maladaptive metacognitive biases, poor emotion regulation and avoidant or emotion‐focused coping contribute to distress, whereas self‐compassion and problem‐focused coping may be protective. (iii) Environmental and contextual factors—traumatic life events, discrimination, daily stressors and substance use amplify distress, often by interacting with internal vulnerabilities and emotion regulation capacities.

**Conclusion:**

Distress in PLEs arises from the interaction between symptom features, internal psychological vulnerabilities and external environmental factors. Consistent with emotion regulation models, distress reflects disruptions in adaptive appraisal and coping processes that heighten emotional reactivity. Conceptualizing distress as a transdiagnostic vulnerability underscores its relevance for early identification and preventive interventions. Future research should harmonise definitions of distress, examine longitudinal pathways and evaluate resilience‐building approaches to mitigate risk and improve mental health outcomes.

## 1 Background

**FIGURE 1:**
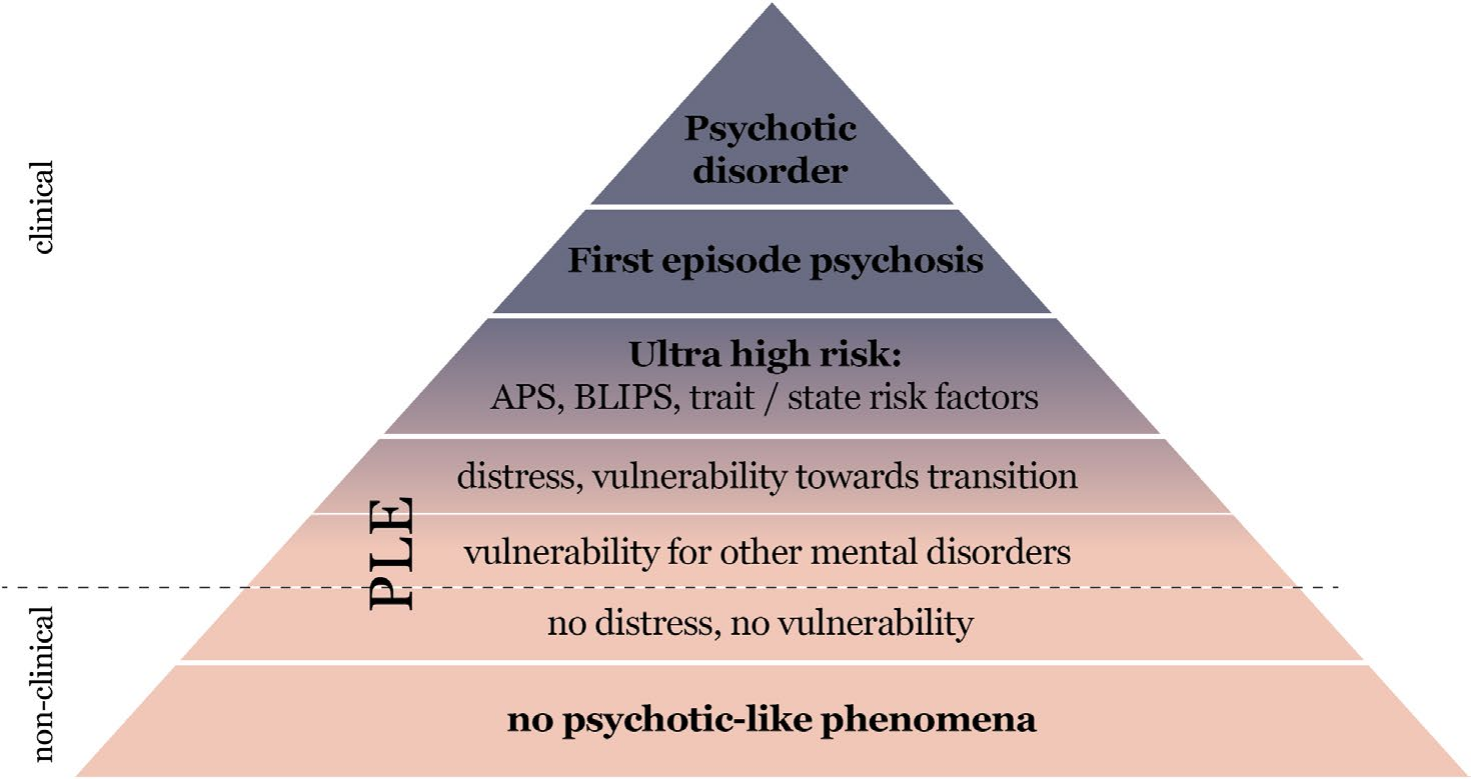
Psychosis continuum modified model (Preti et al. 2012).

Over the past decades, research has increasingly demonstrated that sub‐threshold psychotic experiences do not always indicate a clinical disorder but can also occur in the general population (van Os et al. 2000). This evidence challenges the traditional categorical framework of psychosis and instead supports a spectrum or a continuum model of psychotic phenomena ranging from benign psychotic‐like experiences (PLEs) to full‐blown psychotic symptoms, thereby bridging the gap between clinical and non‐clinical populations (Verdoux and van Os 2002) (see Figure 1).

As a matter of fact, the prevalence of PLEs in the general population is relatively high, though estimates vary depending on assessment methods. Approximately 8% of the general population experience PLEs, with rates peaking during adolescence, when up to a quarter of individuals report such phenomena (Hafeez and Yung 2021; Linscott and van Os 2013; Nuevo et al. 2012; Yung et al. 2009; Sullivan et al. 2020). Despite the absence of a standardised definition (Hinterbuchinger and Mossaheb 2021), PLEs have been conceptualised as ‘psychotic symptoms in the absence of illness’ (Kelleher and Cannon 2011), ‘subclinical psychosis phenotype’ (Linscott and van Os 2010), ‘subthreshold forms of hallucinations and delusions’ (Yung and Lin 2016). They may include subthreshold bizarre experiences, unusual beliefs or magical thinking, persecutory ideas and perceptual anomalies (Linscott and van Os 2013; Prochwicz and Gawęda 2016; Stainton et al. 2021; Yung et al. 2006; Yung et al. 2009).

Most PLEs are infrequent, transient, self‐limiting and non‐distressing, with approximately 80% remitting over time. However, a subset of up to 20% persists, and up to 8% of these cases progress to a psychotic disorder (Hanssen et al. 2005; Linscott and van Os 2013; McGrath et al. 2015; Seiler et al. 2020). In addition to genetic vulnerability, environmental factors such as adverse life events, substance use, socioeconomic disadvantage and urbanicity (Linscott and van Os 2013; Mackie et al. 2011; van Os et al. 2009) have been linked to the persistence of PLEs. Furthermore, longitudinal research suggests that the persistence of PLEs is among the important predictors of transition to clinical psychosis (Dominguez et al. 2011). Cognitive models further propose that how individuals appraise and respond to these experiences may determine whether PLEs become distressing and clinically significant (Peters et al. 2017).

PLEs are not confined to the realm of psychosis, but occur across diagnostic boundaries. For example, they have been reported in up to 27% of individuals with anxiety and affective disorders, supporting the notion that PLEs may represent a transdiagnostic phenotype rather than a psychosis‐specific phenomenon (Wigman et al. 2012; van Os and Reininghaus 2016). This ‘extended psychosis phenotype’ highlights three broad subtypes: (i) psychosis‐specific experiences that indicate vulnerability to psychotic disorders, (ii) incidental or transdiagnostic experiences overlapping with other psychiatric symptoms that indicate vulnerability to other mental disorders (van Os and Reininghaus 2016; Yung et al. 2009; Yung and Lin 2016) and (iii) non‐clinical variants with no clear psychopathological relevance (Yung et al. 2009). Even within non‐clinical cohorts, however, certain subpopulations appear more likely to report PLEs (Grant and Hennig 2020; Hinterbuchinger et al. 2018, 2023). In some of these subclinical samples, PLEs have been linked to impaired psychological functioning and a general vulnerability to psychopathology (Lindgren et al. 2022; Unterrassner et al. 2017), raising the question of why some individuals experience distress while others do not. Importantly, the degree of distress associated with subthreshold psychotic experiences appears to influence their persistence, clinical relevance, and risk of transition to psychosis (Karcher et al. 2022; Nelson and McGorry 2020; Murphy et al. 2018; Dominguez et al. 2011; van Os et al. 2009).

Distress, in psychological terms, refers to a maladaptive emotional or behavioural response to stress, often manifesting as anxiety, depression, anger, fear (Wheaton et al. 2013). Stressors are external or internal challenges that threaten an individual's functioning or well‐being, whereas distress arises when coping and resilience mechanisms are insufficient to manage these challenges (Zeidner and Hammer 1990; Wheaton et al. 2013; Troy et al. 2023). In the context of PLEs—which can be described as an individual stressor—distress can vary widely, from benign curiosity about unusual experiences to significant emotional burden and fear (see Figure 1).

In order to account for the different relevant ways in which distress may be associated with PLEs, we broadened the concept of distress from a mere psychological burden into the following operationalization using two categories: (1) distress directly related to PLEs, typically assessed with validated tools, and (2) distress inferred indirectly through related psychological constructs that can be hypothesised as deriving from distress, including psychiatric comorbidities, suicidality, coping style, emotional dysregulation. This operationalization allowed for a more thorough interpretation and differentiation of the findings with respect to narrowly defined distress attributable to PLEs versus broader indicators of vulnerability to distress in the context of PLEs.

Despite increasing research, it remains unclear which factors directly or indirectly contribute to distress in PLEs, particularly among individuals without a diagnosed mental disorder. Non‐clinical populations offer a unique opportunity to investigate these relationships while minimizing confounding effects of treatment or manifest illness. Moreover, examining protective and risk factors for distress in PLEs among non‐clinical cohorts may provide valuable insights into the continuum hypothesis, inform early intervention strategies and identify resilience mechanisms that prevent the escalation of benign PLEs into clinical conditions. Accordingly, this systematic review aims to synthesise the current evidence on factors associated with distress in psychotic‐like experiences within non‐clinical populations.

## 2 Methods

### 2.1 Literature Search Strategy

This systematic review was conducted in strict accordance with the Preferred Reporting Items for Systematic Reviews (PRISMA) guidelines (Page et al. 2021). To identify relevant studies, a comprehensive electronic database search was conducted, including Medline, Web of Science, Embase and Cochrane. The time frame of the search was from January 1st, 2000 to March 31st, 2025, to ensure comprehensive coverage of relevant literature and to capture recent developments up to the date of the initial search. The search strategy combined MeSH terms for ‘psychotic‐like experiences’ AND ‘distress’ OR ‘psychotic‐like experiences’ AND ‘resilience’ OR ‘psychotic‐like experiences’ AND ‘burden’ OR ‘psychotic‐like experiences’ AND ‘coping’ OR ‘psychotic‐like experiences’ AND ‘adaptive behaviour’. The terms ‘burden’, ‘adaptive behaviour’, ‘coping’ and ‘resilience’ were included in order to account for the semantics closely related to the concept of distress, as described in the introduction.

Two researchers (J.R. and A.R.) independently evaluated titles and abstracts and full texts to determine eligibility based on clearly defined inclusion and exclusion criteria. In addition, a backward search was performed to manually review the references of the selected studies. In the event of disagreement, the following steps were taken: The two researchers independently reassessed the study in question by reviewing their notes and assessment criteria. If discrepancies persisted, the researchers met to discuss their findings and attempted to reach consensus through detailed review and comparison of the study with the inclusion criteria. If consensus could not be reached through discussion, a third researcher (N.M.) was consulted to resolve the discrepancy. This structured approach minimised bias and ensured a rigorous and transparent selection process.

All studies that met the inclusion criteria underwent a quality assessment by two independent researchers (J.R. and A.R.) using the Joanna Briggs Institute Critical Appraisal Checklist for Analytical Cross Sectional studies (Joanna Briggs Institute 2020) and were subsequently confirmed to be of adequate methodological quality. However, we acknowledge heterogeneity in methodological robustness and have indicated in the discussion where findings are based on studies of limited quality or generalizability. Ethical approval was not required for this systematic review as it involved the analysis of published data and did not include any direct interaction with human participants.

### 2.2 Inclusion and Exclusion Criteria

Eligibility for inclusion in our systematic review was defined by the following criteria: We only considered studies involving individuals from the general population, explicitly excluding individuals with help‐seeking behaviour, At Risk Mental State (ARMS), Clinical‐High‐Risk (CHR), Ultra‐High‐Risk for Psychosis (UHR), a history of first‐episode psychosis or diagnosed psychotic disorder. Studies were still included if non‐clinical cohorts were compared to clinical cohorts. In addition, studies had to specifically address PLEs and related distress. All selected articles were required to be peer‐reviewed original publications written in English. We included only studies with a mean participant age above 14 years, as PLEs in younger children often differ in prevalence, development course and clinical implications (Karcher 2022; Kelleher et al. 2012). This restriction enhanced construct validity. Furthermore, studies were excluded if they did not present primary data or if the data were not available in a format suitable for extraction. Studies analysing the same study population were included and accordingly labelled in Table 1.

### 2.3 Synthesis and Analysis of Data

Due to the considerable heterogeneity of variables measured across studies, a meta‐analysis was not feasible. Instead, we conducted a narrative synthesis focusing on potential confounders and mediators identified in the literature. By thematically organizing findings across studies, this approach highlighted how certain variables (e.g., emotional regulation, personality traits) may strengthen or weaken the association between PLEs and distress. This, in turn, helps clarify potential points of intervention and provides a broader context for understanding risk and resilience factors.

**FIGURE 2:**
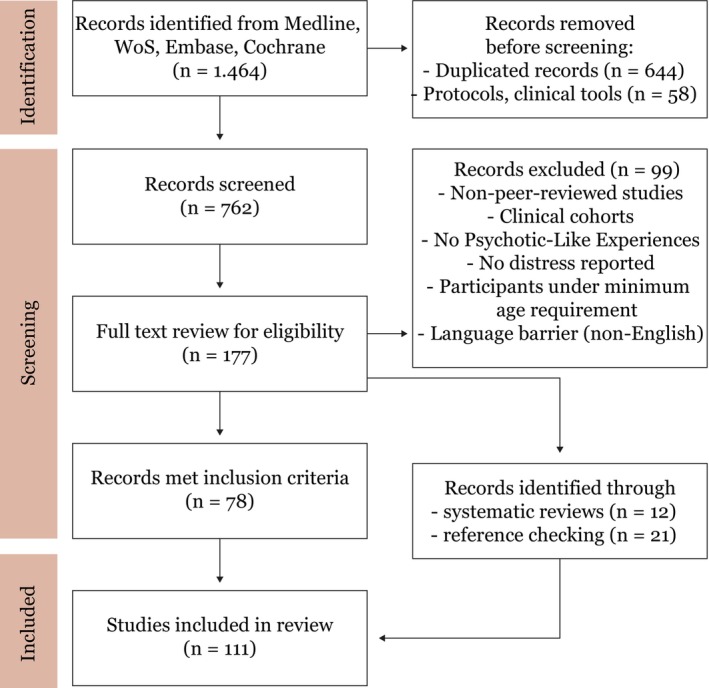
PRISMA 2020 flow chart depicting the different phases of the systematic review process (Page et al. 2021).

## 3 Results

### 3.1 Characteristics of Included Studies

From among a total of 762 articles identified, 78 met the inclusion criteria. An additional 33 studies were identified through a backtrack search as illustrated in Figure 2, bringing the total to 111 studies. The majority of the included studies (87%) used a cross‐sectional study design, while 13% were longitudinal with follow‐up assessments. All of the included original papers used standardised questionnaires; 14 studies also made use of interviews, two were intervention‐based studies and two further studies used qualitative methods. Seven studies (Brett et al. 2009, 2014; Chan et al. 2011; Nolan et al. 2018; So et al. 2015, 2016; Taylor et al. 2014) utilised control cohorts or included individuals with PLEs as a control group in comparison to cohorts with schizotypy, FEP, or manifest psychosis. Data sources were national or international household surveys, birth cohorts, or community samples. In 16 studies, data from eight cohorts were each analysed twice and thus included as study pairs in this review (Brett et al. 2009, 2014; DeVylder, Lukens, et al. 2015; DeVylder and Hilimire 2015; Freeman et al. 2008; Freeman and Fowler 2009; Johnstone, Wong, Girard, and Kim 2024; Johnstone, Wong, Pun, et al. 2024; Koyanagi et al. 2015a, 2015b; Rossi, Socci, et al. 2023; Rossi, Jannini, et al. 2023; Saha, Scott, Johnston, et al. 2011; Saha, Scott, Varghese, and McGrath 2011; Sun et al. 2021, 2023). A range of instruments were used to assess PLEs: The Community Assessment of Psychic Experience (CAPE) (33) was the most commonly used instrument, followed by different versions of the Prodromal Questionnaire (PQ) (23), the Composite International Diagnostic Interview (CIDI) (9), Peters et al. Delusions Inventory (PDI) (9), the Launay‐Slade Hallucination Scale (LSHS) (8), the Appraisals of Anomalous Experiences Interview (AANEX) (4), the Green et al. Paranoid Thought Scales (GPTS) (3), Diagnostic Interview Schedule for Children (DISC) (3), with the rest following.

**TABLE 1:** Overview of included studies.

Authors	Year	Country	*n*	Age (mean, SD)	Female (%)	PLE tool	Primary indicator of distress
Alonso et al.	2018	Global	33 370	—	—	CIDI	Stigma, discrimination
Amendola et al.	2023	Italy	238	24.68 (3.22)	74.8	PQ‐B	Social withdrawal
Andorko et al.	2018	USA	420	20.1 (3.22)	50.6	PQ‐B	Sleep quality
Anglin et al.	2016	USA	644	19.9 (2.11)	66.5	PQ‐92	Discrimination
Armando et al.	2010	Australia	1882	18 (3.5)	65.3	CAPE	Subtype
Bak et al.	2005	Netherlands	36	32.9 (9.8)	58.0	CIDI, BPRS	TLE
Barahmand and Heydari Sheikh Ahmad	2016	Iran	432	21.23 (2.04)	—	PDI, LSHS	Schizotypy
Bernardini et al.	2018	Belgium	257	21.78 (2.71)	66.5	CAPE	Substance use
Boumans et al.	2017	Netherlands	18	44 (19–79)^a^	27.80	CAPE	Coping mechanisms
Bridgwater et al.	2023	USA	2529	20.26 (2.27)	67.0	PRIME	Symptomatology
Brañas et al.	2017	Spain	204	27.18 (5.74)	39.2	CAPE	Sociodemographics
Brett et al.^e^	2009	UK	27^b^/32^c^/56^d^	30.7 (7.54)^d^	41^d^	AANEX	Metacognition
Brett et al.^e^	2014	UK	35^b^/20^c^/36^d^	—	—	AANEX	Appraisal
Campbell and Morrison	2007	UK	544	21.4 (4.4)	67.8	PDI‐B, LSHS‐R	Appraisal
Capra et al.	2015	Australia	1791	22.1 (5.1)	—	CAPE‐P15	Suicidality
Capra et al.	2017	Australia	489	20.8 (−)	81.0	CAPE‐P15	Frequency
Chan et al.	2011	China	4415/536^k^	19.67 (1.00)	34.1	PIC	Schizotypy
Chen et al.	2025	China	4000	20.03 (1.38)	68.8	CAPE‐P15	Sleep quality
Chisholm et al.	2018	Australia, UK	962	16.10 (0.75)^f^/15.51 (0.41)^g^	61.5^f^/54.1^g^	CAPE	Coping mechanisms
Cicero et al.	2019	USA	2738	20.08 (2.51)	72.3	PQ‐B	Sociodemographics
Collip et al.	2013	Belgium	529	27.2 (7.4)	100.0	CAPE, SCID‐I	Daily stress
Cristóbal‐Narváez et al.	2016	Spain	206	21.3 (2.4)	78.6	Individual	TLE
Daalman et al.	2013	Netherlands	72	45.13 (14.5)	72.2	PSYRATS‐AHRS	Metacognition
Dardani et al.	2023	UK	3577	18–24	—	PLIKSi	Neurodiversity
DeVylder and Hilimire^e^	2015	USA	622	18.81 (1.29)	57.8	Individual	Suicidality
DeVylder et al.^e^	2015	USA	590	18.8 (1.56)	60.5	PQ‐B	Suicidality
DeVylder et al.	2013	USA	2434	18–29/30–39/40–49/50–64^h^	49.5	CIDI	Discrimination
DeVylder et al.	2016	Global	176 934	By country	By country	CIDI	Stress sensitivity
Dolphin et al.	2015	Ireland	6062	14.95 (1.62)	51.0	APSS	Coping mechanisms
Ered et al.	2017	USA	454	20.04 (2.31)	73.0	PQ‐92	TLE
Ered et al.	2018	USA	2687	20.22 (3.21)	74.0	PQ‐92	TLE
Fazo et al.	2024	UK, Ireland, France, Germany	706	18.4 (0.6)/22 (0.6)	59.1	CAPE	Adverse‐life events
Fernández et al.	2025	Spain	1824	15.79 (−)	53.8	ESQUIZO‐Q‐A	Transdiagnostic
Fervaha et al.	2015	Canada	370	18.7 (2.3)	71.8	SPQ, individual	Schizotypy, depressive symptoms
Freeman andFowler^e^	2009	UK	200	37.5 (13.3)	50.1	G‐PTS, CAPS	TLE
Freeman et al.^e^	2008	UK	200	37.5 (13.3)	50.0	G‐PTS, CAPS	Metacognition
Freeman et al.	2005	UK	1202	23.0 (6.1)	68.3	PCL, individual	Depressive symptoms
Gawęda et al.	2018	Poland	690	23.30 (3.97)	75.6	PQ‐16, IPASE	TLE, metacognition
Gawęda et al.	2020	Poland	3495	26.39 (4.71)	62.9	PQ‐16	TLE, suicidality
Gawęda and Prochwicz	2015	Poland	110	29.7 (15.5)^i^/27.8 (12.8)^i^	87.3	PDI	Metacognition
Gawęda et al.	2019	Poland	677	22.98 (2.67)	71.1	CAPE, IPASE	Metacognition
Gibson et al.	2019	USA	945	20.13 (2.47)	75.6	PQ‐92	TLE
Gibson et al.	2014	USA	671	20.50 (2.30)	71.0	PQ‐92, SIPS	TLE
Grant and Hennig	2020	Germany	107	22.5 (3.8)	72.0	ESM	Schizotypy
Grattan et al.	2024	New Zealand	466	24.8 (3.6)	58.6	PQ‐B	Discrimination
Hall et al.	2023	USA	727	42.79 (14.42)	66.0	PDI‐21	Discrimination
Haenschel et al.	2023	UK	203	20.2 (3.3)	86.7	CAPE‐P15	Subtype, schizotypy
Hafeez and Yung	2021	UK	590	15.6 (2.64)	53.4	CAPE	Persistence
Honings et al.	2016	Germany, Netherlands	15 837	39.4 (13.8)	53.3	CIDI	Suicidality
Hu et al.	2024	China	4302	19.92 (1.42)	58.9	CAPE‐P8	TLE
Ishigaki and Nishiguchi	2025	Japan	400	20.86 (0.71)	67.8	PCL	General distress, metacognition
Jang et al.	2014	South Korea	6977	16.8 (1.1)	62.8	ESI	Suicidality, depressive symptoms
Johnstone, Wong, Girard, et al.^e^	2024	Canada	300	20.9 (4.4)	78.0	CAPE	Substance use
Johnstone, Wong, Pun, et al.^e^	2024	Canada	351	21.2 (5.6)	82.1	CAPE	Substance use
Karska et al.	2024	Poland	1100	27.1 (5.1)	51.4	PQ‐16	Suicidality
Kline et al.	2012	USA	355	20.3 (2.1)	54.0	PQ‐B	Schizotypy
Kowalski and Gawęda	2022	Poland	840	29.94 (10.39)	72.3	GPTS‐R	Metacognition
Koyanagi et al.^e^	2015a	UK	7403	16–34/35–59/> 60^h^	51.5	PSQ	Non‐suicidal self‐injury
Koyanagi et al.^e^	2015b	UK	7403	16–34/35–59/> 60^h^	51.4	PSQ	Suicidality
Koyanagi et al.	2018	Global	224 842	38.3 (16.0)	50.7	CIDI	Cognitive complaints
Laloyaux et al.	2016	Belgium, Switzerland	112	27.82 (6.33)	43.7	LSHS, PDI	TLE, coping mechanisms
Lee et al.	2019	Korea	1678	18.6 (0.5)	35.8	PQ‐16	Depressive symptoms, anxiety
Liao et al.	2024	China	3060	18.69 (0.89)	66.3	CAPE‐P8	Sleep quality
Lin et al.	2011	Australia	813	15.6 (2.6)	51.0	CAPE	Coping mechanisms
Lindgren et al.	2022	Finland	1313	27.9 (3.6)	54.2	M‐CIDI	General distress
Lindgren and Therman	2024	Finland	157 417	14–16/16–20^h^	52.8	YEAH	Adverse‐life events
Loewy et al.	2007	USA	1020	19.15 (1.4)	66.9	PQ‐92	Frequency
Long et al.	2024	China	2970	18.56 (0.70)	38.9	PQ‐16	Sociodemographics
Lovatt et al.	2010	UK	27	41.4 (10.2)	66.7	AANEX	Appraisal
Luo et al.	2023	China	2231	20.02 (1.39)	69.4	CAPE‐P8	Suicidality
Mamah et al.	2021	Kenya	9564	21.2 (2.0)	46.7	pWERCAP	Severity, neurodiversity
Martin et al.	2015	Australia	1896	14.87 (0.95)	71.6	DISC	Suicidality, general distress
Mętel et al.	2020	Poland	870	26.37 (4.71)	64.0	PQ‐16	TLE, metacognition
Mongan et al.	2019	USA	748	27.93 (4.34)	44.3	PQ‐16	TLE, coping mechanisms
Mylona et al.	2022	Greece	119	14.6 (1.6)	58.0	CAPE	TLE
Nardelli et al.	2024	UK, USA	303	43.94 (14.96)	48.9	CAPE	Emotion regulation
Nishida et al.	2014	Japan	16 131	15.2 (1.7)	51.9	DISC‐C	Suicidality
Nolan et al.	2018	UK	79^d^/26^j^/50^b^	16.2 (1.1)	55.4	APSS	Subtype
Núñez et al.	2021	Chile	302	36.96 (15.5)	60.3	CAPE‐P15	Coping mechanisms
Osborne et al.	2017	USA	234	28.36 (13.26)	62.4	CAPE	Coping mechanisms
Preti et al.	2007	Italy	250	29.9 (9.4)	50.0	PDI	General distress
Preti et al.	2012	Italy	504	24.4 (3.5)	53.6	LSHS, PDI	Certainty
Preti et al.	2014	Italy	649	24 (3.4)	53.0	LSHS	Severity
Prochwicz and Gawęda	2016	Poland	492	21.58 (2.48)	89.4	CAPE	Personality traits
Prochwicz et al.	2020	Poland	290	21.81 (2.72)	86.2	CAPE	Metacognition
Ronald et al.	2014	UK	4743	16.32 (0.68)	55.0	SPEQ	Cognitive disorganization
Rossi et al.^e^	2023	Italy	1010	18.7 (0.65)	49.9	PQ‐16	TLE
Rossi et al.	2021	Italy	500	25.52 (5.84)	71.2	PQ‐16	TLE
Rossi et al.^e^	2023	Italy	1010	18.7 (0.65)	49.3	PQ‐16	TLE, attachment styles
Rössler et al.	2015	Switzerland	1500	29.2 (−)	—	PARA, SIAPA	Subtype
Rössler et al.	2016	Switzerland	663	31.52 (6.77)	52.6	SIAPA, PARA, STS, SNS, CEQ	TLE
Ruzibiza et al.	2018	New Zealand	216	20.0 (2.1)	75.0	SPQ	Stress sensitivity
Saha et al.^e^	2011	Australia	8773	16–85^a^	50.4	CIDI	General distress
Saha et al.^e^	2011	Australia	8841	18–85^a^	50.4	CIDI	Suicidality
Scheunemann et al.	2019	Germany	234	37.0 (15.0)	62.4	PDI, LSHS	Personality traits
Sharifi et al.	2008	Iran	150	23.7 (1.2)	56.0	PDI‐40	Frequency
So et al.	2016	Netherlands	40^b^/261^d^	45.43 (11.95)^b^/50.70 (13.58)^d^	80.0^b^/68.2^d^	LSHS, PSYRATS	Neuroticism
So et al.	2015	China	70^b^/654^d^	20.98 (4.10)	58.4	CAPE	Metacognition
Stainton et al.	2021	UK	302	15.64 (3.29)	57.9	CAPE‐P	Sociodemographics
Sun et al.^e^	2021	China	938	17.65 (0.052)	69.4	CAPE‐P15	Resilience
Sun et al.^e^	2023	China	938	17.65 (0.052)	69.4	CAPE‐P15	Suicidality
Sun et al.	2017	China	9122	14.43 (2.77)	52.3	CAPE	Frequency, TLE
Taylor et al.	2014	UK	20^b^/113^c^/58^d^	22.8 (3.7)	73.3	CAPE, CAARMS	Personality traits
Turley et al.	2019	UK	655	16.6 (.)	—	CAPE	Subtype
Vellante et al.	2012	Italy	437	24.7 (3.5)	59.0	LSHS, PDI	Severity
Vermeiden et al.	2019	Netherlands, Norway, Nigeria	885	23.3 (3.3)	65.1	CAPE‐42	Sociodemographics
Wang et al.	2024	China	4192	16.70 (0.74)	52.7	CAPE	Adverse‐life events
Wu et al.	2022	China	927	23.7 (8.5)	69.4	CAPE‐P15	Cognition
Yung et al.	2009	Australia	875	15.64 (0.46)	52.8	CAPE	Subtype
Zhan et al.	2022	China	2708	17.9 (0.7)	38.7	PQ‐16	TLE
Zhou et al.	2024	Japan	1680	18–20^h^	50.2	DISC	Suicidality

Abbreviations: AANEX = Appraisals of Anomalous Experiences Interview; APSS = Adolescent Psychotic‐like Symptom Screener; BPRS = Brief Psychiatric Rating Scale; CAARMS = Comprehensive Assessment of At Risk Mental States; CAPE = Community Assessment of Psychic Experience; CAPS = Cardiff Anomalous Perceptions Scale; CEQ = Creative Experiences Questionnaire; CIDI = Composite International Diagnostic Interview; DISC = Diagnostic Interview Schedule for Children; ESI = Eppendorf Schizophrenia Inventory; ESM = Experience Sampling Methodology; ESQUIZO‐Q‐A = Oviedo Schizotypy Assessment‐Abbreviated; GPTS = Green et al. Paranoid Thought; IPASE = Inventory of Psychotic‐Like Anomalous Self‐Experiences; LSHS = Launay‐Slade Hallucination Scale; PARA = Paranoia Checklist; PCL. = Paranoia Checklist; PDI = Peters et al. Delusions Inventory; PIC = Paranoid Ideation Checklist; PLIKSi = Psychosis‐Like Symptoms Interview; PQ = Prodromal Questionnaire; PRIME = Prime Screen; PSQ = Psychosis Screening Questionnaire; PSYRATS = Psychotic Symptom Rating Scales; pWERCAP = Washington Early Recognition Center Affectivity and Psychosis; SCID = Structured Clinical Interview for Personality Disorder; SIAPA = Structured Interview for Assessing Perceptual Anomalies; SNS = Schizophrenia Nuclear Symptom Scale; SPEQ = Specific Psychotic Experiences Questionnaire; SPQ = Schizotypal Personality Questionnaire; STS = Schizotypal Signs Scale; TLE = Traumatic Life Experiences; YEAH = Youth Experiences and Health Questionnaire.

^a^ Range.

^b^ Diagnosed.

^c^ UHR/ARMS.

^d^ Non‐clinical.

^e^ Double cohorts.

^f^ UK.

^g^ Australia.

^h^ Age cohorts.

^i^ Low versus high PDI‐score.

^j^ Sexual trauma history or help‐seeking.

^k^ Schizotypal personality disorder.

**FIGURE 3:**
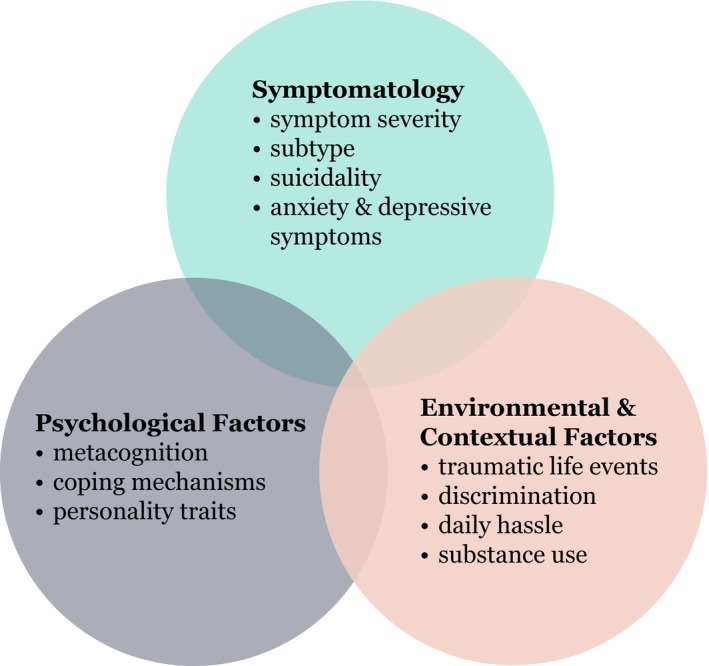
Factors associated with distressing PLEs clustered into symptomatology, psychological factors and environmental influences.

Following our initial search and synthesis, the included studies were categorised by three overarching themes: Symptomatology, Psychological Factors, and Environmental and Contextual Factors (see Figure 3).

Symptomatology refers to the phenomenological characteristics of PLEs, including their frequency, intensity, persistence, and subtype. These factors describe the direct experiential and descriptive features of PLEs and their proximal relationship to distress. To capture the broader affective dimension, findings on co‐occurring anxiety, depression and suicidal ideation are also described here, as symptom‐level indicators that may reflect indirectly inferred distress severity and broader psychopathological burden associated with PLEs.

Psychological factors encompass internal cognitive and affective mechanisms that shape how individuals interpret and regulate their experiences. This category includes metacognitive biases—such as certainty, appraisal and controllability of PLEs—as well as cognitive distortions, personality traits and stress sensitivity. These factors primarily represent indirect pathways through which distress related to PLEs may be amplified or attenuated. In addition, coping strategies are presented as a distinct subtheme, as they represent behavioral and cognitive responses moderating distress linked to PLEs. Together, these factors highlight how appraisal, regulation and resilience processes mediate the subjective impact of PLEs.

Environmental and contextual factors refer to external influences and situational contexts that affect the occurrence of distress associated with PLEs. This includes adverse or traumatic life events, environmental stressors, interpersonal factors such as social functioning and support, and substance use. Discrimination‐related experiences (e.g., racism, stigma or identity‐based bullying) were distinguished from general bullying or victimization, which were categorised as traumatic or adverse life events, depending on context. These external stressors collectively shape indirect vulnerability and resilience in relation to PLE distress.

### 3.2 Symptomatology

#### 3.2.1 Symptom Severity

Several studies report that more frequent PLEs are associated with higher levels of distress (Brañas et al. 2017; Capra et al. 2017; Ered et al. 2017; Lin et al. 2011; Loewy et al. 2007; Mylona et al. 2022; Sun et al. 2017), whereas intermittent or rare PLEs are more commonly observed in non‐clinical populations (Armando et al. 2010). The prevalence of distress directly related to PLEs varies considerably across studies, largely due to differences in measurement tools, definitions and cut‐off criteria. For example, Chan et al. (2011) reported that up to 18% of their sample experienced frequent PLEs, 15%–31% of whom described these as distressing. In contrast, Barahmand and Heydari Sheikh Ahmad (2016) found that 4% of participants reported rare PLEs, with 4.6% rating them as highly distressing. Taken together, the literature consistently indicates that greater frequency, intensity, persistence, and severity of PLEs are associated with increased levels of PLE‐related distress (Campbell and Morrison 2007; Lindgren et al. 2022; Mamah et al. 2021; Preti et al. 2007, 2014; Prochwicz et al. 2020; Saha, Scott, Varghese, and McGrath 2011; Vellante et al. 2012).

#### 3.2.2 Subtypes

Eleven studies examined subtypes of PLEs, most commonly categorised according to the CAPE dimensions. Across these studies, persecutory ideation, paranoid thoughts (Haenschel et al. 2023; Nolan et al. 2018; Ronald et al. 2014), and odd beliefs or bizarre experiences were consistently found to be strongly associated with direct distress. In contrast, perceptual abnormalities, magical thinking and grandiosity showed weaker or more inconsistent associations with distress (Armando et al. 2010; Capra et al. 2017; Turley et al. 2019; Rössler et al. 2015; Yung et al. 2009).

While most studies assessed direct distress resulting from PLEs due to the self‐reporting nature of the instruments used, some also identified indirect psychological distress associated with specific subtypes. For instance, persecutory ideation not only predicts distress directly related to PLEs but also broader affective symptoms such as depression and anxiety, suggesting a shared vulnerability network influencing multiple domains of mental health (Haenschel et al. 2023).

#### 3.2.3 Suicidality

Suicidality demonstrates a bidirectional relationship with PLEs, functioning both as a potential consequence and a contributing factor (Koyanagi et al. 2015a, 2015b; Luo et al. 2023; Saha, Scott, Johnston, et al. 2011; Sun et al. 2023; Zhou et al. 2024; Martin et al. 2015; Nishida et al. 2014). Individuals reporting frequent or distressing PLEs exhibit up to a fivefold increase in the likelihood of suicidal thoughts or behaviours compared to those without such experiences (DeVylder, Jahn, et al. 2015; Gawęda, Pionke, Krężołek, et al. 2020). This association appears particularly pronounced in the presence of comorbid mental disorders (Honings et al. 2016; Jang et al. 2014), exposure to trauma, or reduced resilience (Karska et al. 2024). Certain PLE subtypes—especially persecutory ideation and perceptual abnormalities—have also been linked to elevated suicidal risk and distress (Capra et al. 2015; Gawęda, Pionke, Krężołek, et al. 2020; Sun et al. 2023). Together, these findings suggest a complex, interactive pathway in which PLE frequency, distress intensity, comorbidity and trauma‐related factors jointly amplify vulnerability to suicidality.

#### 3.2.4 Anxiety and Depressive Symptoms

PLEs have been found to predict a broad range of non‐psychotic mental disorders (Lindgren et al. 2022) and are associated with lower perceived mental and physical health (Alonso et al. 2018; Lindgren and Therman 2024). Depressive symptoms and anxiety have been extensively investigated as cofactors influencing the relationship between PLEs and distress. Across studies, PLE subtypes (Freeman and Fowler 2009), persistence (Lin et al. 2011), severity (Dolphin et al. 2015) and both PLE‐associated distress (Brañas et al. 2017; Lee et al. 2019; Mamah et al. 2021) and general psychological distress (Armando et al. 2010; Hafeez and Yung 2021; Haenschel et al. 2023) have been consistently linked to higher levels of depression, anxiety, and negative self‐perception. Additionally, impaired sleep has been repeatedly associated with PLEs (Chen et al. 2025; Liao et al. 2024), with evidence suggesting that this relationship is mediated by rumination, depressive symptoms and traumatic exposure (Andorko et al. 2018; Ered et al. 2018).

Taken together, these findings suggest that depression and anxiety may function both as downstream responses to the distress and occurrence of PLEs and as upstream vulnerability factors contributing to their onset and persistence.

In summary, the degree of PLE‐related and general psychological distress appears to depend less on the mere occurrence of PLEs than on their frequency, intensity and phenomenological characteristics. Moreover, the evidence supports a bidirectional association between PLE‐related distress and broader affective and psychological difficulties.

### 3.3 Psychological Factors

#### 3.3.1 Metacognition

Perceptions and beliefs can be assessed differently depending on an individual's metacognitive bias. Metacognition refers to the ability to reflect on and regulate one's own cognitive processes, including how thoughts and perceptions are evaluated and used to guide behaviour (Flavell 1979). Individual differences in metacognitive knowledge and regulation influence how PLEs are interpreted and the degree of distress they evoke.

PLE‐related distress has been linked to various cognitive and metacognitive biases, including jumping to conclusions and catastrophizing (Freeman et al. 2008; Gawęda et al. 2019; Gawęda and Prochwicz 2015), cognitive disorganization (Ronald et al. 2014), conviction regarding the veracity of unusual experiences (Chan et al. 2011), self‐serving bias—attributing positive events to oneself and negative outcomes to external causes (So et al. 2015)—and maladaptive cognitive beliefs (Brett et al. 2009; Wu et al. 2022). Specific PLEs subtypes, such as paranoid ideation and perceptual abnormalities, have been associated with a jumping‐to‐conclusion bias (Freeman et al. 2008) and, to a lesser extent, emotional reasoning bias (Daalman et al. 2013). Moreover, avoidant safety behaviours and punishment‐ or worry‐based thought control strategies have been shown to predict direct PLE‐related distress (Campbell and Morrison 2007).

Additional metacognitive distortions, such as overestimation of certainty, perceived lack of control and heightened preoccupation or hyperawareness of experiences, are also strongly associated with distress (C. Brett et al. 2014; Bridgwater et al. 2023; Preti et al. 2012).

Beyond their direct effects, these biases may mediate the relationship between PLEs and broader psychological distress, including symptoms of anxiety, depression and somatic dysfunction (Kowalski and Gawęda 2022; Prochwicz et al. 2020). Cognitive distortions such as catastrophizing amplify emotional responses to PLEs (Gawęda and Prochwicz 2015), underscoring the central role of metacognitive processes in shaping both PLE‐related distress and general psychopathology. Indeed, maladaptive metacognitive styles have been associated with poorer overall mental health (Sharifi et al. 2008) and a greater need for clinical intervention (Lovatt et al. 2010).

Individuals reporting subjective cognitive impairments (Koyanagi et al. 2018) or exhibiting autistic traits and communication difficulties also tend to experience higher distress related to PLEs (Mamah et al. 2021; Dardani et al. 2023). Although the mechanisms remain incompletely understood, difficulties in interpreting experiences and heightened sensitivity to internal or social cues may increase anxiety and emotional reactivity, contributing to indirect distress.

In summary, distress in PLEs is shaped less just by the experiences themselves than by metacognitive beliefs and cognitive appraisals that determine how such experiences are interpreted, evaluated and managed. Maladaptive cognitive styles consistently predict greater PLE‐related distress and contribute to a broader psychological burden across both clinical and non‐clinical populations.

#### 3.3.2 Coping Mechanisms

Findings on coping strategies in relation to PLEs are heterogeneous. Qualitative evidence highlights a wide spectrum of interpretative coping mechanisms, ranging from framing PLEs as indicators of mental illness or personal vulnerability to interpreting them as spiritual experiences or protective defences (Boumans et al. 2017).

Quantitative studies similarly reveal that higher PLE scores are associated with greater reliance on avoidant (Dolphin et al. 2015) and emotion focused coping strategies (Freeman et al. 2005), and with reduced use of task‐oriented or problem‐focused coping (Chisholm et al. 2018). Notably, emotion‐focused coping has also been shown to predict the occurrence of PLEs (Lin et al. 2011), suggesting a bidirectional relationship in which maladaptive coping both contributes to and arises from PLE‐related distress.

Individuals experiencing PLEs often report difficulties with emotion regulation (Fernández et al. 2025; Nardelli et al. 2024), particularly reduced habitual acceptance and impaired cognitive and emotional reappraisal (Osborne et al. 2017). Such deficits may originate in early interpersonal environments, such as low parental cohesion, which can hinder emotional development and adaptive regulation capacities (Zhan et al. 2022). These emotional and regulatory vulnerabilities may contribute to functional consequences such as social withdrawal and problematic internet use (Amendola et al. 2023), which can, in turn, amplify distress.

Overall, coping and emotion regulation mechanisms play a central role in shaping how PLEs are experienced and integrated. Early relational and emotional learning factors appear to influence the development of coping styles, which subsequently determine the emotional valence, subjective meaning, and clinical significance of PLEs—potentially transforming otherwise benign experiences into sources of significant distress.

#### 3.3.3 Personality Traits

Personality traits and schizotypal features appear to shape both the emergence and emotional impact of PLEs, underscoring the role of individual vulnerability and resilience profiles. Traits such as self‐transcendence, self‐directedness, persistence, neuroticism and harm avoidance have been identified as predictors of greater PLE‐related distress (Prochwicz and Gawęda 2016; So et al. 2016), whereas self‐compassion is associated with lower PLE frequency and reduced PLE‐related distress (Scheunemann et al. 2019). Individuals reporting distressing PLEs often exhibit lower self‐esteem (DeVylder and Hilimire 2015; Lee et al. 2019), reduced optimism (Dolphin et al. 2015) and negative self‐ and other‐related beliefs (Taylor et al. 2014). Similarly, subclinical negative symptoms, such as amotivation and anticipatory anhedonia, have been linked to heightened PLE‐related distress (Fervaha et al. 2015).

Experiential avoidance—the tendency to suppress or avoid unpleasant internal experiences—has been shown to mediate the relationship between paranoid ideation and symptoms of depression, anxiety and general psychological distress (Núñez et al. 2021). This suggests that maladaptive personality‐related coping tendencies may exacerbate both PLE distress and broader affective difficulties.

Within the schizotypy spectrum, disorganised traits have been associated with increased frequency and conviction of paranoid ideation (Chan et al. 2011), whereas individuals high in positive schizotypy but low in negative and disorganised traits report more PLEs yet experience less PLE‐related distress (Kline et al. 2012), supporting the concept of a resilient or ‘benign schizotypy’ subtype (Grant and Hennig 2020; Kline et al. 2012). In contrast, cognitive‐perceptual schizotypal features, such as magical thinking, suspiciousness and unusual perceptual experiences, are associated with elevated psychological distress, while disorganised and interpersonal features (e.g., social anxiety, emotional constriction) show more variable associations (Barahmand and Heydari Sheikh Ahmad 2016; Ruzibiza et al. 2018).

Taken together, these findings indicate that personality structure and schizotypal traits substantially influence both the frequency and emotional burden of PLEs. While cognitive‐perceptual and maladaptive personality features increase vulnerability to distress in PLEs, positive traits such as self‐compassion, emotional openness and positive schizotypy may serve as protective factors, buffering against adverse outcomes and promoting adaptive integration of unusual experiences.

### 3.4 Environmental and Contextual Factors

#### 3.4.1 Traumatic Life Events

PLEs are closely intertwined with acute and chronic environmental stressors, including traumatic events and discriminatory experiences. According to ICD‐10 (World Health Organization 2019) trauma constitutes a severe stressor ‘of an exceptionally threatening or catastrophic nature, which is likely to cause pervasive distress in almost anyone’. Stress reactivity and impaired resilience appear particularly pronounced among individuals with a history of traumatic life events, with multiple studies demonstrating strong associations between stress sensitivity (Gibson et al. 2014; Rössler et al. 2016), PLE‐related distress (Hu et al. 2024; Mylona et al. 2022; Sun et al. 2021) and exposure to trauma.

These associations are often moderated by psychological and cognitive factors, including maladaptive coping strategies (Ered et al. 2017), rumination (Fazio et al. 2024), depressive symptoms and metacognitive biases such as external locus of control (Freeman and Fowler 2009; Gaweda et al. 2018; Gibson et al. 2019; Laloyaux et al. 2016; Mętel et al. 2020; Mongan et al. 2019; Rossi et al. 2021; Rossi, Jannini, et al. 2023). Within this context, emotional vulnerability and deficits in affect regulation may further intensify both situational and general psychological distress linked to PLEs (Bak et al. 2005; Cristóbal‐Narváez et al. 2016; Lindgren and Therman 2024).

Collectively, these findings indicate that trauma and related environmental stressors heighten stress sensitivity and emotional dysregulation, thereby increasing the likelihood that PLEs become distressing rather than benign experiences.

#### 3.4.2 Discrimination

Discrimination, defined as the ‘unfair or prejudicial treatment of individuals or groups based on characteristics such as race, ethnicity, or sexual orientation’ (American Psychological Association 2024), has been consistently associated with higher rates of PLEs (Dolphin et al. 2015; Alonso et al. 2018; Dolphin et al. 2015; Anglin et al. 2016). Individuals with minority status—including ethnic and sexual minorities—show increased vulnerability to PLEs (DeVylder and Hilimire 2015; Cicero et al. 2019), a relationship likely mediated by acculturative stress (DeVylder et al. 2013), low social support (Brett et al. 2014), and experiences of bullying (Wang et al. 2024).

However, not all minority groups exhibit elevated distress in the presence of PLEs; some studies have found no significant increase in distress among certain ethnic minority populations (Grattan et al. 2024; Hall et al. 2023), suggesting that cultural, social, or other protective factors may buffer against the adverse effects of discrimination. Furthermore, findings from a global survey indicate that while cultural background does not universally predict PLE prevalence or distress, PLEs themselves can elicit stigma and discriminatory responses within social environments (Vermeiden et al. 2019; Alonso et al. 2018).

Together, these results highlight the complex interplay between discrimination, minority stress and social context in shaping both the occurrence and distress associated with PLEs.

#### 3.4.3 Daily Hassle

Micro‐level interactions with one's environment—commonly referred to as daily hassles (Wheaton et al. 2013)—have been shown to interact closely with PLEs. Heightened daily stress reactivity and interpersonal strain arising from routine social interactions (Ishigaki and Nishiguchi 2025) are associated with both increased PLE prevalence (DeVylder et al. 2016; Mamah et al. 2021) and greater persistence of experiences over time (Collip et al. 2013; DeVylder and Hilimire 2015).

Although women tend to report higher levels of daily stress, findings regarding sex differences in the prevalence of PLEs and their associated distress remain inconsistent across studies (Brañas et al. 2017; Lindgren et al. 2022; Lindgren and Therman 2024; Long et al. 2024; Vermeiden et al. 2019; Stainton et al. 2021).

#### 3.4.4 Substance Use

Substance use, particularly when involving cannabis and psychedelics, has been consistently associated with greater frequency and severity of PLEs (Brañas et al. 2017; Bernardini et al. 2018; Johnstone, Wong, Girard, and Kim 2024). Notably, cannabis use, when combined with frequent and distressing PLEs, has been linked to poorer executive functioning, suggesting potential additive or synergistic effects between substance use and cognitive vulnerability (Johnstone, Wong, Pun, et al. 2024).

In summary, converging evidence indicates that environmental adversity—including trauma, discrimination, daily stressors and substance use—is closely linked to an increased subjective burden of PLEs. These findings underscore the importance of understanding distressing PLEs within the dynamic interaction between an individual and their environment, rather than viewing them as isolated or purely intrinsic phenomena.

## 4 Discussion

To our knowledge, this is the first systematic review to comprehensively examine distress in psychotic‐like experiences in non‐clinical populations by integrating both direct distress related to the experiences themselves and indirect distress arising from broader psychological and environmental factors.

Synthesizing 111 studies, our findings demonstrate that distress in PLEs is a multifactorial phenomenon, shaped by an interplay of symptom characteristics, psychological processes and environmental contexts.

Overall, three key insights emerge:

1. Symptom‐level features such as frequency, persistence and subtype (especially persecutory ideation) are strongly associated with higher distress.
2. Internal psychological factors, including metacognitive biases, emotion regulation difficulties and personality traits, influence how PLEs are appraised and managed.
3. External contextual factors—such as trauma, discrimination, daily stress and substance use—further modulate distress, often through their impact on stress sensitivity and coping.

Together, these findings support a dynamic, transdiagnostic model in which distress arises from the interaction between individual vulnerability and environmental stress exposure, rather than from PLEs themselves in isolation.

### 4.1 Internal Vulnerability: The Role of Appraisal, Emotion and Personality

Our results corroborate that the way individuals interpret and regulate PLEs largely determines their emotional impact. Maladaptive metacognitive biases (e.g., catastrophizing, overestimation of threat or lack of control) and poor emotion regulation amplify distress by heightening affective reactivity and sustaining negative appraisal cycles (Cristóbal‐Narváez et al. 2016; Daalman et al. 2013; Gawęda and Prochwicz 2015; So et al. 2016). This supports models proposing a self‐reinforcing loop between cognition and emotion: maladaptive appraisals intensify emotional arousal, which in turn biases further cognitive interpretation, perpetuating distress and functional impairment.

Individual personality traits—particularly neuroticism, harm avoidance and low self‐compassion—emerge as stable vulnerability factors that shape this process (Prochwicz and Gawęda 2016; Scheunemann et al. 2019). Conversely, positive schizotypy and self‐compassion may act as protective traits, promoting flexible interpretation and integration of PLEs. These person‐centred differences explain why similar experiences can be benign for some but highly distressing for others.

### 4.2 External Factors: Environmental Stress and Adversity

Environmental factors further modulate distress through stress sensitization and emotion regulation pathways. Traumatic life events have a particularly robust association with both the presence of and distress linked to PLEs (Gibson et al. 2014; Mylona et al. 2022; Hu et al. 2024; Gawęda, Pionke, Hartmann, et al. 2020). Trauma may alter salience processing and increase threat anticipation, thereby intensifying the emotional valence of unusual experiences. Similarly, discrimination and acculturative stress contribute to PLE‐related distress via mechanisms of chronic social threat and reduced social support (Anglin et al. 2016; Brett et al. 2014).

Daily stressors—'micro‐level hassles’ that accumulate over time—also correlate with both PLE prevalence and persistence (DeVylder et al. 2016; Collip et al. 2013). While women tend to report greater daily stress, sex differences in distress remain inconsistent, indicating that individual stress reactivity rather than gender itself may be the more relevant factor. Additionally, substance use, especially cannabis, may exacerbate distress through additive effects on cognitive control and affective dysregulation (Johnstone, Wong, Girard, and Kim 2024).

These findings collectively illustrate that external adversity and contextual stress act not only as triggers but also as amplifiers of internal vulnerability, underscoring the need to conceptualise PLE distress within a broader psychosocial framework.

### 4.3 Interactions Between Internal and External Factors

Distress in PLEs emerges through reciprocal interactions between internal vulnerabilities and external stressors. Cognitive biases, emotion dysregulation and neurotic traits can heighten sensitivity to environmental challenges, which, in turn, exacerbate psychological reactivity and maintain maladaptive appraisal loops. This aligns with Gross's (2015) emotion regulation model, where distress arises from difficulties across multiple regulatory stages—situation selection, attentional deployment and cognitive reappraisal.

In this view, PLEs are not distressing by default; rather, distress reflects dysregulated emotion regulation processes that fail to mitigate the affective impact of anomalous experiences. Experiences involving threat, loss of control or identity disruption are particularly prone to evoking intense distress (Kapur 2003; Miyata 2019). Importantly, resilience factors—such as adaptive coping, flexible emotion regulation and social connectedness—can interrupt this cycle, fostering adaptive integration rather than escalation. Strengthening these mechanisms may therefore constitute protective leverage points for early intervention.

### 4.4 Coping and Resilience as Protective Mechanisms

While vulnerability factors are well‐established, far less attention has been paid to protective mechanisms that may buffer against distress. Across studies, task‐oriented and problem‐focused coping styles are associated with lower distress, whereas emotion‐focused and avoidant strategies predict increased vulnerability (Dolphin et al. 2015; Chisholm et al. 2018). Early relational environments characterised by emotional support and cohesion appear to foster adaptive coping, reducing the likelihood that unusual experiences become threatening or pathological (Zhan et al. 2022).

Resilience—defined as the ability to maintain or regain psychological stability following stress—may thus mediate whether PLEs remain transient and benign or escalate into persistent distress. Self‐compassion, acceptance, and cognitive flexibility may be key resilience components that transform PLEs from sources of fear into tolerable or even meaningful phenomena. As a matter of fact, studies in clinical populations have examined the efficacy of interventions specifically targeting distress regulation, suggesting that psychological and skill‐based interventions are associated with a reduction in distress and an improvement in coping (Lawlor et al. 2022; Mei et al. 2021). In non‐clinical populations, self‐compassion has been associated with lower distress (Scheunemann et al. 2019), suggesting that resilience‐related processes may represent targets for psychotherapeutic interventions even at subclinical levels of psychosis.

### 4.5 Distressing PLEs and Psychosis‐Risk Trajectories

Consistent with previous work, the prevalence of distressing PLEs in non‐clinical populations varies widely across studies due to methodological heterogeneity and differing definitions of distress (van Os et al. 2009). While many PLEs are transient and non‐distressing and no definitive boundary separates benign from clinically relevant PLEs (Rössler et al. 2015), distress consistently appears as a marker of severity, of persistence and of need for clinical care (Linscott and van Os 2013; Yung et al. 2009). As a matter of fact, greater levels of distress have been shown to predict poorer health outcomes over time (Barry et al. 2020). While frequent and distressing PLEs appear to align with increased psychosis risk in the general population (Loewy et al. 2007), some researchers caution against relying solely on self‐reported PLEs as a marker for clinical risk (Nelson and Yung 2009; Yung and Lin 2016). For example, Schultze‐Lutter et al. (2014) argue that in PLEs, actual psychotic symptom prevalence is often overestimated, especially when unaccompanied by distress or dysfunction. Longitudinal findings further complicate this picture: Sullivan et al. (2020) report that approximately 60% of individuals who develop psychosis experienced PLEs years prior, yet 85% did not meet operationalised ultra‐high risk for psychosis criteria at initial presentation and 30% did not seek help at all. These findings support the proneness–persistence–impairment model (van Os et al. 2009), in which PLEs become clinically relevant only when persistent and distressing. Standardised clinical interviews such as the Comprehensive Assessment of At‐Risk Mental States (CAARMS; Yung et al. 2005) or Structured Interview of Psychosis‐risk Syndrome (SIPS; Miller et al. 2003), which assess operationalised criteria for at‐risk mental states, may help mitigate diagnostic uncertainty.

However, distress should not be interpreted as a deterministic predictor of psychosis. Rather, it may serve as a nonspecific marker of underlying psychological strain and emotion dysregulation. Indeed, many individuals who experience distressing PLEs never develop a psychotic disorder but may remain vulnerable to other affective or anxiety‐related difficulties. Thus, distress may indicate general psychopathological liability rather than a linear psychosis trajectory, emphasizing the importance of early identification and support focused on distress reduction.

### 4.6 Limitations

Despite the integrative synthesis achieved in this review, several methodological limitations must be acknowledged. Most included studies employed cross‐sectional designs with self‐report instruments, limiting causal inference and longitudinal interpretation. The specific search terms used may have excluded studies employing alternative terminology for PLE; however, the aim was to specifically address the concept of psychotic‐like experiences. Heterogeneity in study design, quality of included studies, and methodological rigour, as well as assessment tools and definitions of distress, further complicate comparability across findings, thereby impacting the overall conclusions drawn from the review. Nevertheless, this variability also offers valuable insight into the transdiagnostic relevance of PLE distress across settings and populations and allows for the identification of patterns, thus offering insights into how different approaches to assessing PLE and distress might reveal broader trends or underlying factors.

### 4.7 Conclusion

This systematic review highlights that distress in psychotic‐like experiences arises from the interaction between internal psychological vulnerabilities and external environmental factors, rather than just from the experiences themselves. Our findings emphasise that distress functions as a critical marker of psychological vulnerability and may guide both early intervention and preventive strategies. Strengthening emotion regulation, coping flexibility and resilience capacities could mitigate distress and reduce the likelihood of progression toward clinical psychosis or other mental disorders.

Future research should aim for a greater harmonization of assessments and definitions and focus on longitudinal trajectories and intervention studies that explicitly target distress regulation within the psychosis continuum. By shifting attention from the mere presence of PLEs to the emotional meaning and management of these experiences, clinicians and researchers are better able to differentiate benign, adaptive phenomena from those signalling risk of clinical need.

## Funding

The authors have nothing to report.

## Data Availability

Data sharing not applicable to this article as no datasets were generated or analysed during the current study.
